# Multi-scenario based robust intensity-modulated proton therapy (IMPT) plans can account for set-up errors more effectively in terms of normal tissue sparing than planning target volume (PTV) based intensity-modulated photon plans in the head and neck region

**DOI:** 10.1186/1748-717X-8-145

**Published:** 2013-06-18

**Authors:** Martin Stuschke, Andreas Kaiser, Jehad Abu Jawad, Christoph Pöttgen, Sabine Levegrün, Jonathan Farr

**Affiliations:** 1 Department of Radiotherapy, University Hospital Essen, Essen 45147, Germany; 2 West German Proton, Therapy Centre Essen, Essen 45147, Germany; 3 Current address: Department of Radiologic Sciences, St. Jude Children's Research Hospital, Memphis TN, USA

**Keywords:** Intensity modulated proton therapy, IMPT, Robust optimization, Head and neck cancer, Re-irradiation

## Abstract

**Background:**

In a previous report, we compared the conformity of robust intensity-modulated proton therapy (IMPT) plans with that of helical tomotherapy plans for re-irradiations of head and neck carcinomas using a fixed set-up error of 2 mm. Here, we varied the maximum set-up errors between 0 and 5 mm and compared the robust IMPT-plans with planning target volume (PTV) based intensity-modulated photon therapy (IMRT).

**Findings:**

Seven patients were treated with a PTV-based tomotherapy plan. Set-up margins of 0, 2, and 5 mm were subtracted from the PTV to generate target volumes (TV) TV_0mm_, TV_2mm_, and TV_5mm_, for which robust IMPT-plans were created assuming range uncertainties of ±3.5% and using worst case optimization assuming set-up errors of 0, 2, and 5 mm, respectively. Robust optimization makes use of the feature that set-up errors in beam direction alone do not affect the distal and proximal margin for that beam. With increasing set-up errors, the body volumes that were exposed to a selected minimum dose level between 20% and 95% of the prescribed dose decreased. In IMPT-plans with 0 mm set-up error, the exposed body volumes were on average 6.2% ± 0.9% larger than for IMPT-plans with 2 mm set-up error, independent of the considered dose level (p < 0.0001, F-test). In IMPT-plans accounting for 5 mm set-up error, the exposed body volumes were by 11.9% ± 0.8% smaller than for IMPT-plans with 2 mm set-up error at a fixed minimum dose (p < 0.0001, F-test). This set-up error dependence of the normal tissue exposure around the TV in robust IMPT-plans corresponding to the same IMRT-plan led to a decrease in the mean dose to the temporal lobes and the cerebellum, and in the D2% of the brain stem or spinal cord with increasing set-up errors considered during robust IMPT-planning.

**Conclusions:**

For recurrent head and neck cancer, robust IMPT-plan optimization led to a decrease in normal tissue exposure with increasing set-up error for target volumes corresponding to the same PTV.

## Background

The consideration of a 2 mm set-up error seems adequate for small targets in the head and neck region when daily online navigation is used [1,2]. However, in the head and neck region, less frequent online correction protocols and off-line protocols correcting only systematic set-up errors may require margins between the clinical target volume (CTV) and the planning target volume (PTV) accounting for larger set-up errors of 5 mm or more [3,4].

Robust intensity-modulated proton therapy (IMPT) treatment plan optimization, optimizing the dose distribution in the worst case of multiple scenarios covering possible realizations of set-up errors and density variations, can result in treatment plans that maintain target volume coverage and normal tissue sparing in the presence of set-up errors and range uncertainties [5-10]. A single scenario PTV based treatment planning may not be sufficient for IMPT because possible spatial deviations of dose gradients between spots can affect dose homogeneity within the PTV with set-up error [5,11].

In a previous study, we compared dose conformity and normal tissue exposure of intensity-modulated photon plans for helical tomotherapy with robust IMPT-plans for set-up errors of 2 mm [6]. Here, we test the performance of the robust multi-scenario based IMPT optimization to spare normal tissues around a given PTV per patient for different set-up errors varying between 0 and 5 mm. With increasing set-up errors, the CTV-PTV set-up margin contains an increasing portion of the PTV and robust IMPT optimization based on the CTV may lead to results which depend systematically on the set-up margin size.

## Findings

### Methods

#### Dataset

The analysis was based on 7 patients with recurrent head and neck carcinomas who were re-irradiated using helical tomotherapy. Patients and tomotherapy treatment planning were described in a previous publication [6].

#### Intensity-modulated proton therapy planning

Target volumes TV_0mm_, TV_2mm_, and TV_5mm_ were obtained from the original tomotherapy PTV of each patient by concentric shrinkage by 0, 2, and 5 mm, respectively. IMPT-plans were generated to cover TV_0mm_, TV_2mm_, and TV_5mm_ using the field configurations described in the previous study [6]. Robust optimization was used assuming a range, i.e. density, uncertainty of +/− 3.5%. In addition, maximum set-up errors of 0, 2, and 5 mm in each direction were considered in the robust optimization of the IMPT-plans based on TV_0mm_, TV_2mm_, and TV_5mm_, respectively. IMPT planning was performed using the RayStation v2.4.13.31 planning system as described by Fredrikson et al. [7]. Robust optimization evaluates the penalties of the original plan and the plans with shifted beam isocenters corresponding to the maximum set-up errors and altered density scalings. Optimization of the pencil beam spot weights is performed to minimize the penalties of the worst case scenario. In a previous publication, IMPT-plans assuming set-up errors of 2 mm and using TV_2mm_ as CTV were compared to tomotherapy plans optimized on the PTV, i.e. TV_0mm_[6]. In the present study, we compared, for every patient, the robust IMPT-plans based on TV_0mm_, TV_2mm_, and TV_5mm_, assuming maximum set-up errors of 0, 2, and 5 mm, respectively (IMPT_0mm_, IMPT_2mm_, and IMPT_5mm_, respectively) with the original PTV-based tomotherapy photon plan. Differences in the dose distributions of the IMPT_0mm_-, IMPT_2mm_-, and IMPT_5mm_-plans reflect the performance of a robust scenario-based optimization to handle set-up errors in comparison to a PTV-based approach.

#### Statistical analysis

The effect of the set-up error on the body volume exposed to doses higher than x% of the prescribed dose (V_body_x%), where 20% ≤ x% ≤ 95%, in the IMPT-plans IMPT_0mm_, IMPT_2mm_, and IMPT_5mm_, respectively, was analyzed using Proc Glm, SAS statistical software Version 9.2 (Cary, NC). The logarithm of the ratio of V_body_x% exposed in plan IMPT_ymm_ to V_body_x% exposed in plan IMPT_2mm_ (i.e. Log(V_body_x% in IMPT_ymm_ / V_body_x% in IMPT_2mm_) was used as dependent variable, where ymm denotes a set-up error of y mm. The set-up error was used as an independent classification variable, the individual patient as a random variable, and the dose level x and the square of the dose level x·x as continuous regressor variables. Dose differences to organs at risk in the IMPT_ymm_-plans compared with the IMPT_2mm_-plans were analyzed in a similar manner.

## Results

### Exposed body volumes

**Figure 1:**
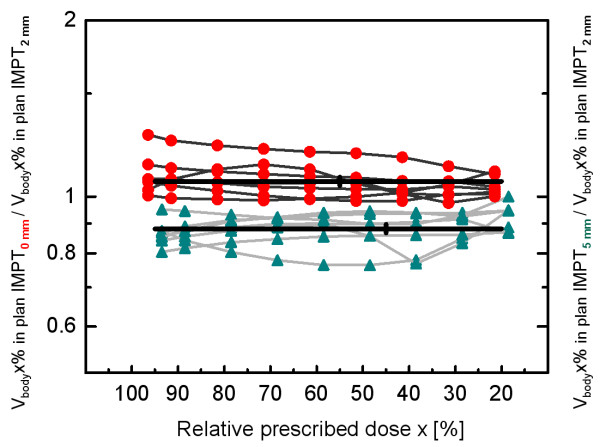
**Exposed body volumes.** Body volumes exposed to doses higher than x% of the prescribed dose (V_body_x%) in robust IMPT-plans assuming set-up errors of 0, 2, and 5 mm around the target volume derived from the original PTV through isotropic shrinkage by set-up margins of 0, 2, and 5 mm, respectively, for 7 patients with recurrent head and neck carcinomas and evaluated isodose levels x between 20% and 95%. The ratios of V_body_x% in the 0 and 5 mm IMPT-plans (IMPT_5mm_ and IMPT_0mm_, respectively) over V_body_x% exposed in the 2 mm IMPT-plan (IMPT_2mm_) are displayed by red circles and green triangles, respectively. A significant decrease of −11.9 ± 0.8% in V_body_x% in the robust IMPT_5mm_-plan was obtained in comparison with V_body_x% exposed in the robust IMPT_2mm_-plan (p < 0.0001, F-Test). The respective increase in V_body_x% in the IMPT_0mm_-plan was 6.2 ± 0.9% (p < 0.0001, F-Test).

For all evaluated isodose levels x between 20% and 95%, V_body_x% exposed in the robust 5 mm plans (IMPT_5mm_) was on average smaller (mean: -11.9%, 95% confidence interval of the mean: -13.4% to −10.3%, p < 0.0001, F-test) than V_body_x% exposed in the robust 2 mm plans (IMPT_2mm_). Compared with the IMPT_2mm_-plans, a 6.2% increase of the exposed body volume V_body_x% was obtained in the 0 mm plans (IMPT_0mm_), averaged over all patients and isodose levels (95% confidence interval: 4.4% to 8.1%, p < 0.0001, F-test). The deviations of the body volumes exposed in the IMPT_5mm_- and IMPT_0mm_-plans from the body volume exposed in the IMPT_2mm_-plan did not depend on the selected isodose level x, because neither a linear nor a quadratic term of x became significant using a Taylor series expansion (p > 0.25 for each term). Figure 1 shows the ratios of V_body_x% exposed in the IMPT_5mm_- and IMPT_0mm_-plans over V_body_x% exposed in the IMPT_2mm_-plan for all 7 patients and all evaluated isodose levels x between 20% and 95%. There was a significant inter-patient variability in the mean increase of the exposed body volume for the IMPT_0mm_-plans compared with the IMPT_2mm_-plans over V_body_x% (p < 0.0001, F-Test). The smallest increase amounted to 1.2% (patient 6), the largest increase was 19.0% (patient 1) (mean increase: +6.2%). The inter-patient variability in the decrease of the exposed body volume for the IMPT_5mm_-plans compared with the IMPT_2mm_-plans was also significant (p < 0.0001, F-Test). The smallest decrease was −6.9% (patient 6), the largest decrease amounted to −18.6% (patient 1) (mean decrease: -11.9%). Average depths of the proximal and distal CTV border at the isocenter were 3.7 cm and 8.6 cm, respectively, over all coplanar fields for the considered patients with larger inter-field variations. The corresponding range uncertainties were 1.3 mm and 3.0 mm in this study.

**Figure 2:**
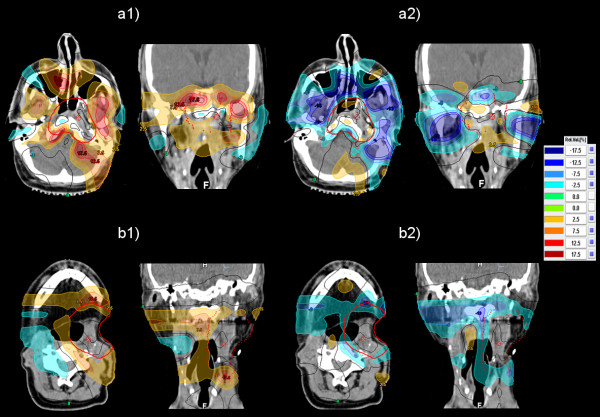
**Dose difference plots.** Distribution of the dose difference obtained by subtracting the dose distribution of the IMPT_2mm_-plan from the dose distributions of the IMPT_0mm_- and the IMPT_5mm_-plans. (a1) Dose difference IMPT_0mm_-IMPT_2mm_ for patient 1. (a2) Dose difference IMPT_5mm_-IMPT_2mm_ for patient 1. The corresponding dose difference plots for patient 7 are shown in (b1) and (b2), respectively.

Figure 2 shows dose difference plots calculated by subtracting the dose distribution of the IMPT_2mm_-plan from the dose distribution of the IMPT_0mm_- and IMPT_5mm_-plans for patients 1 and 7, respectively. These dose difference distributions demonstrate the increase of the dose around the target volume in the 0 mm IMPT-plan and the decrease of the dose in the 5 mm IMPT-plan compared with the 2 mm IMPT-plan. On average, the TV_0mm_-size was 20% larger and the TV_5mm_-size 37% smaller than the size of the TV_2mm_. The absolute values are given in [6].

The coverage of the robust IMPT-plans for 0, 2, and 5 mm set-up errors remained high with respect to the relevant target volumes TV_0mm_, TV_2mm_, and TV_5mm_. In the IMPT_0mm_-plans, the dose to 95% of the target volume was >98% of the prescribed dose for patients 1 and 6 and >100% of the prescribed dose for all other patients. In the IMPT_2mm_- and IMPT_5mm_-plans, the dose to 95% of the target volume was >95% of the prescribed dose for patient 1 and >100% for all other patients. The robustness against diagonal set-up errors was tested for the 2 and 5 mm IMPT-plans by shifting the plan isocenter by 1.5 or 3.5 mm in the lateral and ventro-dorsal directions and applying a density change of +3%. The IMPT_5mm_-plans were as robust against combined set-up errors of 3.5 mm in the directions specified above as the IMPT_2mm_-plan against set-up errors of 1.5 mm.

### Normal tissue exposure

**Figure 3:**
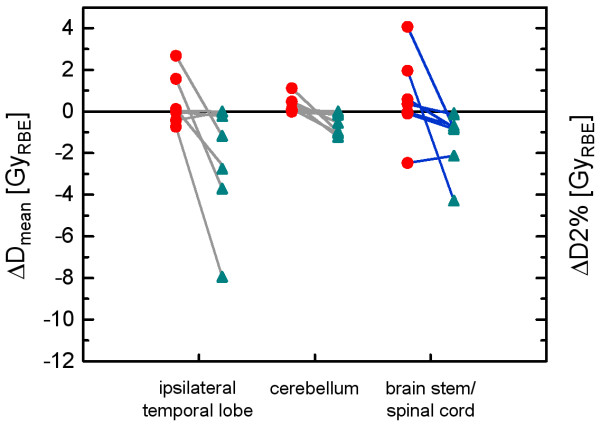
**Normal tissue exposure.** Mean dose difference to the ipsilateral temporal lobe and the cerebellum between the IMPT_0mm_- and IMPT_2mm_-plans (red circles) and between the IMPT_5mm_- and IMPT_2mm_-plans (green triangles) for the 7 patients (left vertical axis). The respective differences in D2%, quantifying hot spots in the serial organ of each patient exposed to the highest doses, i.e. the either brain stem or the spinal cord, are also shown (right vertical axis).

Dose difference distributions between the IMPT_0mm_- and the IMPT_2mm_-plans and between the IMPT_5mm_- and the IMPT_2mm_-plans were also analyzed with respect to the exposure of selected normal tissues and organs at risk. Differences in the mean doses to the ipsilateral temporal lobe and to the cerebellum in the IMPT_0mm_- and IMPT_5mm_-plans compared with the IMPT_2mm_-plan are shown in Figure 3 for the 7 patients. The mean doses to the ipsilateral temporal lobe and to the cerebellum showed a dependence on the size of the set-up error considered in the robust optimization (p = 0.009 and p = 0.001, F-tests). Smaller mean doses were obtained for larger set-up errors considered. The same holds for the D2%-differences in the brain stem and spinal cord between the IMPT_0mm_- and the IMPT_5mm_-plans compared with the IMPT_2mm_-plan (Figure 3). Considering the serial organ with the highest D2% in each patient, either the brainstem or the spinal cord, the D2%-difference to the IMPT_2mm_-plan was significantly larger for the IMPT_0mm_-plan compared with the IMPT_5mm_-plan (p = 0.016, F-test).

## Discussion

Set-up margins alone cannot ensure dose coverage of the CTV in IMPT-plans with multiple fields and high in-field dose gradients [5,11]. Deviations in the position of these dose gradients in the patient from spot to spot due to set-up errors or range uncertainties can result in under- or overdosage inside the PTV. Robust IMPT optimization can lead to smooth dose gradients per field across a target volume and can diminish the risk of pencil beams stopping directly in front of an abutting normal tissue [5,8,9]. It makes use of the feature that set-up errors in beam direction alone do not affect the distal and proximal margin for that beam as also a beam-specific PTV-margin concept would do [12] but unlike the PTV concept of expanding the CTV by set-up margin used in photon therapy. Scanned particle beams can spare normal tissues in beam direction behind the target volume and conform to the proximal contour of the target volume so that a reduction of normal tissue exposure per beam can result. Unlike CTV expansion by set-up margins, the robust IMPT optimization method also considers the potential influence of lateral set-up errors on the water equivalent depth of the distal and proximal CTV border at a given lateral spot position due to lateral density inhomogeneities in the entrance channel, e.g. if bone is moved in front of the target volume at that spot position due to set-up error. Multi-scenario based robust IMPT-plans can maintain dose coverage of the target volume better than PTV-based IMPT-plans [5,13]. On the other hand, the spatial dose distribution can be considered as independent of small set-up errors and organ motions for rotational or fixed-field intensity-modulated photon therapy [14]. Thus, the PTV concept is usually adopted to account for set-up errors in intensity-modulated photon therapy. In this study, we showed that for recurrent head and neck carcinomas robust IMPT-plans perform the better relative to a photon intensity-modulated plan the larger the set-up margin as a part of the PTV is. This holds true for highly constrained IMPT field arrangements with up to 7 fields. Therefore, with increasing set-up margin portion of the PTV due to larger set-up errors, robust optimization can increase the on average lower conformity of IMPT in comparison with helical tomotherapy at or above the 50% isodose found in the previous study [6] for 2 mm set-up errors. Multiple scenarios, considered in robust optimization could also by linked by elastic deformations in the future to simulate internal motion and body deformation. The magnitude of range uncertainties assumed might also vary slightly from institution to institution [15].

## Conclusion

Unlike the PTV-approach for photon IMRT, robust multi-scenario based IMPT optimization can increasingly reduce the normal tissue exposure around the target volume for recurrent head and neck cancer with increasing set-up margin portion of the PTV.

## Competing interests

Parts of this work were supported by Grant No. STU-151/9-1 from the German Research Foundation (Deutsche Forschungsgemeinschaft). The authors declare that they have no competing interests.

## Authors’ contributions

MS carried out the plan comparisons, performed the statistical analysis and drafted the manuscript. He also contributed to the optimization of both helical tomotherapy and proton therapy plans. AK performed the robust optimization and evaluation of the IMPT-plans. He reviewed and edited draft versions of the paper. JAJ and CP conducted clinical treatment of the patients, generated initial volumes of interest, and approved the clinical helical tomotherapy plans. SL performed the helical tomotherapy optimization and the dosimetric analysis of both tomotherapy and IMPT-plans. She also reviewed and edited draft versions of the paper. JF was involved in improving the proton therapy optimization approaches and in the design of the proton therapy planning system. He also participated in concept discussions for developing the investigative aims and critically reviewed the draft manuscript. All authors read and approved the final manuscript.
